# Uncoupling Protein 2 in Cardiovascular Health and Disease

**DOI:** 10.3389/fphys.2018.01060

**Published:** 2018-08-02

**Authors:** Xiao Yu Tian, Shuangtao Ma, Gary Tse, Wing Tak Wong, Yu Huang

**Affiliations:** ^1^School of Biomedical Sciences, Institute of Vascular Medicine, Li Ka Shing Institute of Health Sciences, The Chinese University of Hong Kong, Hong Kong, China; ^2^Division of Nanomedicine and Molecular Intervention, Department of Medicine, Michigan State University, East Lansing, MI, United States; ^3^Department of Medicine and Therapeutics, Li Ka Shing Institute of Health Sciences, The Chinese University of Hong Kong, Hong Kong, China; ^4^School of Life Sciences, The Chinese University of Hong Kong, Hong Kong, China

**Keywords:** uncoupling protein 2, cardiovascular disease, endothelial function, mitochondria, oxidative stress

## Abstract

Uncoupling protein 2 (UCP2) belongs to the family of mitochondrial anion carrier proteins. It uncouples oxygen consumption from ATP synthesis. UCP2 is ubiquitously expressed in most cell types to reduce oxidative stress. It is tightly regulated at the transcriptional, translational, and post-translational levels. UCP2 in the cardiovascular system is being increasingly recognized as an important molecule to defend against various stress signals such as oxidative stress in the pathology of vascular dysfunction, atherosclerosis, hypertension, and cardiac injuries. UCP2 protects against cellular dysfunction through reducing mitochondrial oxidative stress and modulation of mitochondrial function. In view of the different functions of UCP2 in various cell types that contribute to whole body homeostasis, cell type-specific modification of UCP2 expression may offer a better approach to help understanding how UCP2 governs mitochondrial function, reactive oxygen species production and transmembrane proton leak and how dysfunction of UCP2 participates in the development of cardiovascular diseases. This review article provided an update on the physiological regulation of UCP2 in the cardiovascular system, and also discussed the involvement of UCP2 deficiency and associated oxidative stress in the pathogenesis of several common cardiovascular diseases. Drugs targeting UCP2 expression and activity might serve another effective strategy to ameliorate cardiovascular dysfunction. However, more detailed mechanistic study will be needed to dissect the role of UCP2, the regulation of UCP2 expression, and the cellular responses to the changes of UCP2 expression in normal and stressed situations at different stages of cardiovascular diseases.

## Introduction: Uncoupling Protein and the Regulation of Mitochondrial ROS

When ATP is produced from mitochondria, electrons flow from NADH or FADH_2_ to molecular oxygen (O_2_) through complexes I to IV of the respiratory chain along with the oxidation of nutrient substrates. This leads to protons being pumped from the mitochondrial matrix into the intermembrane space, generating a proton-motive force consisting of both voltage (ΔΨ_m_) and concentration gradients. **Uncoupling proteins** (UCP1/2/3) are the major proteins responsible for inducing proton leak (Echtay et al., 2002; Brand and Esteves, 2005). UCP1 is abundant and exclusively expressed in brown adipose tissue. UCP2 and UCP3 share very similar amino acid sequence with UCP1. UCP1 evolved in mammals as an adaptive trait for cold acclimatization via thermogenesis. UCP2 is ubiquitously expressed in most cell types and tissues, while UCP3 is less abundantly expressed, and can be found in muscle, heart, and brown adipose tissue (Fleury et al., 1997; Samec et al., 1998; Damon et al., 2000; Sokolova and Sokolov, 2005). The biochemical properties of U have been reviewed in details elsewhere (Bouillaud et al., 2016).

Reactive oxygen species (ROS) derived from the mitochondria (mitoROS) represent a major source of oxidative stress within the cell (Meany et al., 2006). Two major sites of mitoROS generation are complex I (by flavin mononucleotide in the presence of NADH) (Orr et al., 2013) and III (electron transfer from ubiquinol to cytochrome c) (Giorgio et al., 2005; Guzy et al., 2005). UCPs, especially UCP2 maintains ROS at an acceptable level, while UCP1 only buffers ROS in the thermo-neutral condition (Oelkrug et al., 2014). The uncoupling function of UCPs to eliminate ROS requires an activator, which is the superoxide itself, when interacting with UCPs, causes rise of proton conductance (∼proton leak), in the presence of fatty acids (Echtay et al., 2002). Disturbance of the antioxidant system, such as cellular damage, can result in the generation of mitoROS, of which a large proportion is O_2_^∙^, which is converted into H_2_O_2_ by superoxide dismutase in the mitochondria. H_2_O_2_ is more stable and can travel freely through the cell, participating in signaling transduction. However, excessive amount of H_2_O_2_ is harmful, as it can oxidize thiol residues of different proteins, including kinases, transcription factors, and enzymes, thereby altering their functions (Groeger et al., 2009). Although many enzymes such as glutathione peroxidase and catalase have the ability to scavenge H_2_O_2_, uncoupling remains a necessary mechanism because of its ability to reduce or minimize changes during respiration and thereby controlling mitoROS levels within the cell. Upregulation of mitochondrial fatty acid uptake, associated with higher mitochondrial membrane potential and low ATP level, can also significantly increase ROS production (Seifert et al., 2010).

The uncoupling function and ROS-scavenging mechanism of UCP2 and UCP3 has been well studied by experiments that alter the expression or activation of UCP2/3 in different models. The mild uncoupling theory for controlling ROS levels was first identified by experiments demonstrating UCP2 inhibition causes a rapid increase of H_2_O_2_ (Negre-Salvayre et al., 1997). In Ucp2^-/-^ mice, increased mitoROS production is observed, which aids phagocytosis of infectious agents such as bacteria in macrophages (Arsenijevic et al., 2000). Similar findings have been observed in Ucp3^-/-^ mice exhibiting excessive mitoROS production, with no change in other metabolic processes such as thermogenesis or fatty acid oxidation (Vidal-Puig et al., 2000; Mailloux et al., 2012a).

However, others have argued that UCP2 is not a uncoupler under physiological conditions (Bouillaud, 2009; Shabalina and Nedergaard, 2011; Diano and Horvath, 2012). For example, a report on mitochondrial bioenergetics comparing Ucp2^+/+^ and Ucp2^-/-^ genotypes found no difference in proton leaks between these systems (Couplan et al., 2002). In addition, ATP/ADP ratio is lowered in the presence of an uncoupler protein, but it was not increased in the lungs or spleens of Ucp2^-/-^ mice. Furthermore, UCP2 expression levels in mitochondria of spleen and kidney were significantly different, yet similar degrees of superoxide-induced uncoupling was observed. Instead, UCP2 only regulates ROS levels by a metabolic switch or triggered by a stimulus, for instance, ROS *per se* (Bouillaud, 2009). Regardless of the mechanism, the ability of UCPs, especially UCP2, to reduce oxidative stress makes them an attractive therapeutic target in cardio-metabolic and neurodegenerative disorders, in which excessive ROS production plays a key role in their disease pathogenesis.

## Transcriptional Regulation of UCP2

**FIGURE 1:**
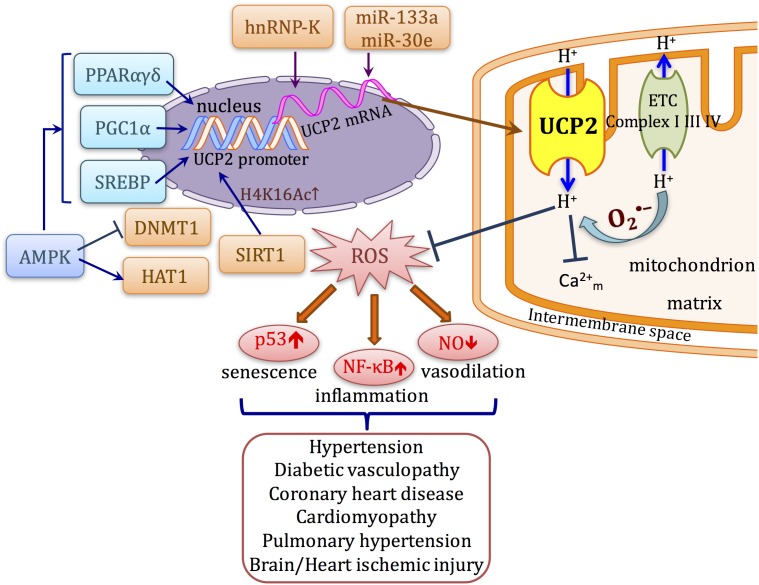
Schematic diagram describing the transcriptional regulation and function of UCP2 in cardiovascular system. UCP2 uncouples oxygen consumption with ATP synthesis. ROS activates the proton conductance of UCP2 and also lowers proton-motive force. Inhibition of ROS production attenuates the upregulation/activation of several signaling pathways such as NF-κB and p53 that lead to cellular senescence, inflammation, and impaired vasodilation, which occur as common mechanisms in a variety of cardiovascular diseases. The transcription of UCP2 can be directly affected by transcription factors such as PPARs, SREBPs, and PGC1α. Meanwhile, UCP2 expression can also be modulated at post-transcriptional and post-translational levels by different mechanisms.

Because of its important function to regulate cellular mechanism, the expression of UCPs in response to metabolic stress, nutrient availability, and immuno-modulators is tightly regulated at different levels including transcriptional regulation by nuclear receptors facilitated by fatty acids, translational control at the 5′ UTR, and post-translational protein degradation by lysosomal activity. In this review, we focus on the discussion of the regulation of UCP2 expression and function, particularly in the cardiovascular system (**Figure 1**).

### Regulation by PPARs

The nuclear receptors PPARγ and PPARδ regulate UCP2 gene expression at the transcriptional level. Although analysis of the Ucp2 promoter found no direct PPAR response elements, several regulatory elements, including (i) SP1 motif, (ii) sterol response element (SRE) that can be recognized by SREBPs, and (iii) a E-Box-like motif that mediates PPARδ responsiveness of the UCP2 promoter, both at basal or when activated by PPARδ ligand (Medvedev et al., 2001), possibly through interaction with other transcription factors. For instance, PPARδ works together with KLF5 to bind to the Ucp2 promoter in mouse. PPARδ agonist increases de-SUMOylation of KLF5 to promote this transactivation, thereby working together to govern fatty acid metabolism in the heart, muscle, and liver (Oishi et al., 2008). PPARα activation also results in increased UCP2 expression. Natural ligands of PPARα, either free fatty acids or synthetic ligand WY-14643 up-regulate UCP2 in mouse hearts (Murray et al., 2005). However, the study using mtDNA mutator mice (transgenic mice that has dysfunction of respiratory chain components and premature aging without increasing oxidative stress) shows that UCP2 does not alter ROS production or proton leak, but is responsible for maintaining fatty acid oxidation. Loss of UCP2 causes accumulation of lipid contents in the cardiomyocytes, associated with increased expression of glycolytic gene and a consequent increase in lactic acid levels (Kukat et al., 2014). This indicates a significant role of UCP2 to mediate the adaptive response of mitochondria on the plasticity of nutrient utilization, especially when mitochondrial respiration is deficient, which has not been well explored.

In the liver, PPARα activation induces UCP2 expression which lowers mitochondrial H_2_O_2_ production during fatty acid oxidation, through decreasing the levels of fatty acyl-carnitines, to preserve liver function in drug-induced hepatotoxicity (Patterson et al., 2012). Conversely, UCP2/3 expression in adipocytes can be regulated by PPARγ, which was later revealed to be a direct binding motif of PPARγ to intron of Ucp3 that also interacts with Ucp2 gene through structural change (Bugge et al., 2010). Similar indirect transactivation mechanism is also responsible for PPAR-induced UCP2 expression in pancreatic β cells and is involved in glucose-stimulated insulin secretion (Oberkofler et al., 2009; Jiang et al., 2010).

### Regulation of UCP2 by Other Pathways

The Ucp2 promoter contains a SRE region. In pancreatic β-cells, overexpression of the master regulator of lipogenesis, SREBP1c, upregulates UCP2 expression, which is activated by free fatty acids (Medvedev et al., 2002; Yamashita et al., 2004). UCP2 also mediates several effects of another important protein kinase in energy metabolism, AMPK. AMPK activator AICAR upregulates UCP2, suppresses ROS production, and inhibits palmitate-induced apoptosis in endothelial cells (Kim et al., 2008). AMPK-induced UCP2 promotes angiogenesis in mouse model of hind limb ischemia. In AMPKα1 and α2 KO mice, angiogenesis, eNOS activity, and UCP2 expressions are all impaired (Xu et al., 2011). AMPK-UCP2 pathway is down-regulated in stroke-prone spontaneously hypertensive rats, associated with earlier progression of hypertension and renal injury (Rubattu et al., 2015a). Our group demonstrated the protective effects of GLP-1 ligand exendin-4 was mediated by a similar mechanism (Liu et al., 2014). This AMPK-UCP2 pathway is also responsible for the inhibitory effects of ghrelin (Wang et al., 2010) and endurance training (Calegari et al., 2011) on insulin secretion. The upregulation of UCP2 by AMPK is likely to be attributed to PPARα, which directly induces UCP2 transcription. Similar to UCP1, UCP2 can also be induced by cAMP/PKA which modulates mitochondrial Ca^2+^ level and UCP2 transcription. The loss of this PKA-UCP2 pathway causes mitochondrial dysfunction and excessive ROS generation, leading to endothelial dysfunction of coronary arterioles in mice (Xiong et al., 2016). Taken these evidences into consideration, UCP2 activation can be triggered by metabolic stress, especially free fatty acids, as a feedback to modulate ROS level, which makes it important for maintaining homeostasis especially under stress such as exercise or hyperlipidemia, in organs such as heart, skeletal muscle, etc.

## Translational and Post-Translational Regulation of UCP2

### Regulation of UCP2 by RNA-Binding Protein

UCP2 mRNA translation is constantly suppressed and UCP2 protein has a very short half-life which is rapidly degraded by ubiquitin-proteasome system, comparing to UCP1 (Azzu and Brand, 2010). However, the implication of UCP2 degradation in cardiovascular diseases is not fully understood. In cells such as macrophages, UCP2 protein level was altered despite unchanged mRNA expression, suggesting post-transcriptional or translational regulation was also present. One study suggested that the RNA-binding protein heterogeneous nuclear ribonucleoprotein-K (hnRNP-K) is involved in post-transcriptional control of human UCP2 mRNA in endothelial cells. hnRNP-K binds to specific mRNA including human UCP2, and its function is directly controlled by tyrosine kinase Src to induce phosphorylation (Ostrowski et al., 2004). Angiopoietin-1 acts via Src to release UCP2 mRNA from hnRNP-K, thereby rapidly increasing UCP2 protein expression. This provides the explanation for the rapid response of UCP2 to oxidative stress in endothelial cells (Tahir et al., 2014). However, whether this mechanism also contribute to angiopoietin-1 function in maintaining endothelial integrity while reducing permeability and inflammation is not studied. In addition, similar stabilization of mouse Ucp2 mRNA by hnRNP-K has been observed in mouse liver. Adiponectin, an adipocyte-derived cytokine, can translocate hnRNP-K and subsequently increases UCP2 protein expression and has been implicated in protection against fatty liver (Zhou et al., 2012).

### Other Mechanisms Involved in UCP2 mRNA Translation

Besides transcriptional and post-transcriptional regulation, UCP2 protein can be regulated by glutathionylation, in which GSH is conjugated to cysteine residues. UCP2/3 is glutathionylated and remains inactive when mitoROS level is low. Increased levels of mitoROS can turn on GSH to induce deglutathionylation and subsequent activation of UCP2/3 (Mailloux et al., 2011). This reversible process is responsible for the regulation of ROS on UCP2-mediated inhibition of glucose-stimulated insulin secretion in pancreatic β-cells (Mailloux et al., 2012b).

UCP2 mRNA contains a long 5′ UTR and an upstream ORF within the 5′ UTR that has an inhibitory role on UCP2 translation (Hurtaud et al., 2006). Interestingly, glutamine, among all the amino acids, can increase UCP2 protein expression, and this effect is likely due to enhanced translation efficiency stimulated by glutamine through re-initiation on the UCP2 coding sequence after translation of the short upstream ORF (Hurtaud et al., 2007). Glutamine can therefore modulate protein expression and upregulate UCP2, thus to inhibit glucose-induced insulin secretion.

### Epigenetic Regulation of UCP2 Expression

The discrepancy between UCP2 protein and mRNA expression in tissues such as heart and skeletal muscle also indicates a potential role of microRNA regulation. A muscle-specific miRNA, miR-133a directly recognizes the 3′-UTR of mouse Ucp2 mRNA. MyoD directly binds to upstream regions of miR-133a during myogenesis associated with the suppression of Ucp2, which prevents differentiation (Chen et al., 2009). A later study showed that miR-133a and its suppression on Ucp2 are also involved in the increase of caspase-1 p10 and Il1b p17 cleavage, thus promotes inflammasome activation in monocytes and macrophages (Bandyopadhyay et al., 2013). This illustrates the regulatory role of UCP2 in enforcing ROS-mediated inflammatory response (Bandyopadhyay et al., 2013). Ucp2 is also a direct target of miR-30e. TGF-β1 induces Ucp2 expression and miR-30e through inhibition of UCP2 lessens TFG-β1-induced epithelial-mesenchymal transition. Ucp2^-/-^ mice are protected from renal fibrosis (Jiang et al., 2013). In high salt-treated stroke-prone spontaneously hypertensive rats, UCP2 is downregulated, accompanied by increase of rno-miR-24 and miR-34a, which are known to directly target UCP2 in the kidney (Di Castro et al., 2013). The regulation of UCP2 by miRNAs provides some new targets in the diseases affecting liver, heart, muscle, and β cells.

UCP2 expression is also regulated through nucleosome remodeling. In human endothelial cells, the mechanism by which AMPK upregulates UCP2 as well as UCP2 involves demethylation by inhibition of DNMT1 and histone acetylation induced by HAT1, which are both phosphorylated by AMPK (Marin et al., 2017). In neurons, UCP2 upregulation under hypoxia is accompanied by histone deacetylation at H4K16Ac, and upregulation of SIRT1 (Dmitriev and Papkovsky, 2015). Skeletal muscle ischemia induces myokine irisin which upregulates UCP2 expression in the lung against oxidative stress induced by ischemic injury (Chen et al., 2017). Similarly, in endothelial cells, UCP2 is upregulated by SIRT1 mediated deacetylation (Olmos et al., 2013), unlike in pancreas SIRT1 downregulates UCP2 expression (Bordone et al., 2006).

## Role of UCP2 in the Cardiovascular System

### UCP2 Inhibits Oxidative Stress in Endothelial Cells

Oxidative stress is the major culprit of dysfunction of endothelial and vascular smooth muscle cells and contributes to the pathogenesis of vascular disorders. Because UCP2 is one of the major antioxidant proteins in the mitochondria to keep ROS level in control, it has attracted the attention of cardiovascular research scientists since its discovery. UCP2 function has been studied in various cell types of the cardiovascular system, including endothelial and smooth muscle cells, cardiomyocytes, monocytes and macrophages. In an acute inflammatory response, ROS production in macrophages is required for the defense against invading microorganisms, which can be dampened in macrophage without UCP2. However, during low-grade chronic inflammation, in conditions such as atherosclerosis, ROS produced by vascular macrophages can exert deleterious effects on the arterial wall including cell adhesion and migration, which can be reversed by UCP2 overexpression (Ryu et al., 2004). In endothelial cells, most of the mitoROS are produced during state 4 respiration. Studies on the isolated mitochondria from bovine aortic endothelial cells demonstrate that UCP2 overexpression induces proton leak without altering basal or antimycin-A (complex III)-induced superoxide production, probably because UCP2 is not activated during complete inhibition of respiration (Fink et al., 2005).

Subsequently, it was shown in human aortic endothelial cells that UCP2 overexpression slows LPC- or linoleic acid-induced ROS (mainly H_2_O_2_ and ONOO^-^) production and NF-kB activation. UCP2 also restores state 3 and state 4 respiration, which is impaired by LPC or linoleic acid (Lee et al., 2005). In retinal endothelial cells and pericytes, UCP2 expression increases along with glucose gradient to accommodate ROS production induced by glucose (Cui et al., 2006). Suppression of UCP2 changes mitochondrial membrane potential and reduces ATP and glycolysis, which is important for proliferative and angiogenic functions of endothelial cell, particularly by regulating endothelial surface molecules such as CD34 and CD105, resulting in premature senescence. This phenotype can be rescued by inhibition of ROS-sensitive p53, indicated by decreased senescence marker p16, p21, and β-gal staining, suggesting that p53 acts as a downstream effector of UCP2 ablation on endothelial cell senescence (Shimasaki et al., 2013) (**Table 1**). More details on the critical role of UCPs in cardiovascular disease have been discussed in a recent review by another group (Cheng et al., 2017).

**Table 1:** The function of UCP2 in cells associated with vascular cells.

Cell types	Roles	References
Endothelial cells	↓ Oxidative stress and ↓ apoptosis,	Lee et al., 2005; He et al., 2014; Li et al., 2014; Rubattu et al., 2017b
	↓ Mitophagy,	Haslip et al., 2015
	↓ Senescence,	Li et al., 2014
	↑ Angiogenesis	Xu et al., 2011
Vascular SMCs	↓ Oxidative stress, ↓ ER stress, and	Dromparis et al., 2013
	↓ proliferation	
	↓ Oxidative stress	Ma et al., 2014a
	↓ Mitochondrial dysfunction and	Pak et al., 2013
	↓ proliferation	
	↓ Proliferation, ↓ migration, and	Park et al., 2005
	↓ PAI expression	
	↓ Proliferation	Zhang et al., 2017 (Park et al., 2005; Dromparis et al., 2013; Pak et al., 2013; Ma et al., 2014a; Zhang et al., 2017)
Cardiomyocytes	↓ Oxidative stress, ↓ apoptosis,	Bodyak et al., 2007;
	↓ apoptosis, and ↓ hypertrophy	Li et al., 2014
	↓ Hypertrophy	Wang et al., 2018
	↑ Tolerance to hypoxia and reoxygenation	Hang et al., 2007
	↑ Tolerance to hypoxia and	Ma et al., 2014b; McLeod et al., 2005
	↓ apoptosis	
	↓ Mitochondrial dysfunction, and	Quan et al., 2018
	↓ excitation–contraction coupling,	
	↓ Apoptosis	Turner et al., 2010
Macrophages	↓ Accumulation and ↓ apoptosis	Blanc et al., 2003
	↑ Anti-oxidative capacity	Moukdar et al., 2009
	↓ Adhesion and ↓ migration,	Ryu et al., 2004
	↓ ROS production and	
	↑ thermogenesis	Van De Parre et al., 2008

Apart from ROS, whether UCP2 acts as a sensor of other mitochondrial metabolic substrates in vascular cells is unclear. As we described in earlier paragraph, upregulation of UCP2 in myocardium in response to hypoxia alleviates ischemia insults (Quan et al., 2018), which is similar to what has been observed in neurons. As regards to its regulation of cellular metabolism, UCP2 in cancer cells and stem cells favors oxidative metabolism and AMPK activation. UCP2 also inhibits glucose oxidation in mitochondria and promotes glutaminolysis (Vozza et al., 2014). Whether such metabolic reprogramming function of UCP2 is also important for endothelial cells in sensing metabolites such as fatty acid, glucose, lactate, etc., especially in context such as vasculogenesis when cells are exposed to nutrient gradient, remains to be explored.

### UCP2 and Vascular Dysfunction

The effect of UCP2 on oxidative stress in vascular cells has been extended to vasomotor functional studies. We and others have shown that UCP2 overexpression in isolated rat or mouse aortic segments protects endothelium-dependent nitric oxide-mediated dilatation that is impaired by cardiovascular risk factors such as high glucose, oxidized LDL (LPC), and angiotensin II (Lee et al., 2005; Tian et al., 2012; Liu et al., 2014). Ucp2^-/-^ further increases high glucose-stimulated ROS elevation and deteriorates the impaired dilatation in mouse aortas, which can be rescued by adenoviral overexpression of Ucp2. We demonstrated that in the context of high fat diet (HFD)-induced obesity, Ucp2^-/-^ further impairs nitric oxide-dependent vasodilation, while UCP2 overexpression *in vivo* rescues obesity-related endothelial dysfunction (Tian et al., 2012). A more recent study showed similar result that loss of UCP2 exacerbates endothelial dysfunction of coronary arteries from mice on HFD. In their model, after feeding with HFD, the expression of UCP2 is induced as a protective feedback, which is dependent on cAMP and PKA activation. Loss of UCP2 causes excessive ROS generation and further impairs vasodilation in HFD fed mice (Xiong et al., 2016). In summary, studies of UCP2 on endothelial cells demonstrate the anti-oxidant and anti-inflammatory role of UCP2 that can become a potential target of vascular dysfunction associated with excessive ROS production (**Table 1**).

### UCP2 and Hypertension

The expression of UCP2 is lower in skeletal muscle of hypertensive rats when compared with normotensive rats (Fukunaga et al., 2000). In human subjects, the *UCP2*-866A allele is more frequent in hypertensive patients than normotensive subjects (Ji et al., 2004). Later study found that transcriptional upregulation of UCP2 by rosiglitazone attenuates oxidative stress associated neurogenic hypertension induced by microinjection of angiotensin II into the rostral ventrolateral medulla, indicating that UCP2 is involved in the central regulation of blood pressure (Chan et al., 2009). Using the Ucp2^-/-^ mice, we found that Ucp2 ablation augments high salt diet-induced hypertension and vascular dysfunction (Ma et al., 2010). Ucp2^-/-^ further raises the superoxide level and attenuates nitric oxide-dependent dilatation of resistance arteries, thus propagating hypertension.

To further dissect the cell types important in UCP2 inhibition-induced hypertension, we generated transgenic mice with vascular smooth muscle-specific overexpression of UCP2 protein by expressing exogenous smooth muscle myosin heavy chain promoter driven human UCP2 gene (Ma et al., 2012). These transgenic mice are protected against salt-induced hypertension, in line with what has been found in Ucp2^-/-^ mice, suggesting that UCP2 plays a significant part in the regulation of vascular salt sensitivity (Lombard, 2010). In addition, UCP2 ablation exacerbates the high salt diet-induced cardiovascular and renal fibrosis in the ROS-dependent manner (Ma et al., 2014b). Other groups confirmed that UCP2 participates in high salt diet-induced target organ damage as well (Di Castro et al., 2013; Rubattu et al., 2015a,b,c). More recent study showed that in the brain endothelial cells of stroke prone spontaneous hypertensive rats, microRNA-503 downregulates UCP2, contributing to stroke occurrence in response to high salt diet (Rubattu et al., 2017b). In addition, high salt also led to UCP2 downregulation, susceptibility to oxidative stress and exacerbated renal damage in this rat model (Rubattu et al., 2017a). These data suggest that UCP2 function against oxidative stress is important in protecting vascular function and thus alleviates high blood pressure and hypertension-related secondary organ damage, representing an attractive target for the treatment of hypertension and its complications (**Table 2**).

**Table 2:** The effect of UCP2 expression in various pathologies of the cardiovascular system.

Disease/Pathology	Roles	References
Pulmonary hypertension in mice	- Reduces apoptosis and mitophagy in endothelial cells - Inhibits oxidative stress, ER stress, inhibits smooth muscle proliferation - Mitochondrial dysfunction	Haslip et al., 2015; Dromparis et al., 2013 Pak et al., 2013 (Lee et al., 2005; Xu et al., 2011; Tian et al., 2012; He et al., 2014; Li et al., 2014; Wang et al., 2014; Haslip et al., 2015; Rubattu et al., 2017b)
Stroke	↓ Apoptosis in endothelial cells ↑ Angiogenesis	Rubattu et al., 2017b
Endothelial dysfunction	↓ Senescence in endothelial cells ↓ Oxidative stress in endothelial cells	Lee et al., 2005; Wang et al., 2014 Tian et al., 2012
High salt induced hypertension	- Inhibits oxidative stress in smooth muscle cells - Reduces renal damage - Reduces cardiac remodeling	Ma et al., 2014a Ma et al., 2014b Ma et al., 2014b
Neointimal hyperplasia	- Inhibits vascular smooth muscle hyperplasia	Zhang et al., 2017
Ventricular hypertrophy	- Reduces apoptosis in cardiomyocytes	Hang et al., 2007
Doxorubicin-induced cardiotoxicity	- Reduces oxidative stress and apoptosis in cardiomyocytes	Wang et al., 2018
Atherosclerosis	- Reduces macrophage accumulation in plaque, reduces apoptosis - Increases anti-oxidative capacity of macrophage - Reduces adhesion and *trans*-endothelial migration of monocytes	Blanc et al., 2003 Moukdar et al., 2009 Ryu et al., 2004
Pulmonary hypertension in mice	- Reduces apoptosis and mitophagy in endothelial cells	Haslip et al., 2015;
	- Inhibits oxidative stress, ER stress, inhibits smooth muscle proliferation	Dromparis et al., 2013
	- Mitochondrial dysfunction	Pak et al., 2013 (Lee et al., 2005; Xu et al., 2011; Tian et al., 2012; He et al., 2014; Li et al., 2014; Wang et al., 2014; Haslip et al., 2015; Rubattu et al., 2017b)
Stroke	↓ Apoptosis in endothelial cells	Rubattu et al., 2017b
	↑ Angiogenesis	Xu et al., 2011
Endothelial dysfunction	↓ Senescence in endothelial cells	Lee et al., 2005; Wang et al., 2014
	↓ Oxidative stress in endothelial cells	Tian et al., 2012
High salt induced hypertension	- Inhibits oxidative stress in smooth muscle cells	Ma et al., 2014a
	- Reduces renal damage	Ma et al., 2014b
	- Reduces cardiac remodeling	Ma et al., 2014b
Neointimal hyperplasia	- Inhibits vascular smooth muscle hyperplasia	Zhang et al., 2017
Ventricular hypertrophy	- Reduces apoptosis in cardiomyocytes	Hang et al., 2007
Doxorubicin-induced cardiotoxicity	- Reduces oxidative stress and apoptosis in cardiomyocytes	Wang et al., 2018
Atherosclerosis	- Reduces macrophage accumulation in plaque, reduces apoptosis	Blanc et al., 2003
	- Increases anti-oxidative capacity of macrophage	Moukdar et al., 2009
	- Reduces adhesion and *trans*-endothelial migration of monocytes	Ryu et al., 2004

### UCP2 and Atherosclerosis

Although ROS in macrophage helps to defend again pathogen insults, excessive ROS production in chronic low-grade inflammation which is common in cardiovascular disease is detrimental to the tissue. The protective effect of UCP2 has also been studied in atherosclerosis. The first study used bone marrow transplantation from Ucp2^-/-^ and Ucp2^+/+^ mice into Ldlr^-/-^ mice followed by atherogenic diet. UCP2 expression can be detected in mitochondria from splenic cells of Ldlr^-/-^ mice receiving Ucp2^+/+^ bone marrow cells. Analysis of plaque composition reveals increases in macrophage population, nitrotyrosine expression, apoptotic cell percentage, and collagen content in Ucp2^-/-^ transplanted mice, while the adaptive immune response and smooth muscle phenotype are unaltered, comparing to Ucp2^+/+^ transplanted mice, suggesting that the reduction of plaque coverage is mainly due to UCP2 in macrophage (Blanc et al., 2003). The effect of UCP2 on macrophage metabolism and thermogenesis may be involved (Van De Parre et al., 2008). Ucp2^-/-^ mice can develop atherosclerotic plaque (although minimal comparing to Apoe^-/-^) when fed with atherogenic diet (Moukdar et al., 2009). The expression of UCP2 in vascular smooth muscles also contributes to the inhibition of oxidative stress in response to vascular injury, and protects against neointimal formation associated with pro-inflammatory NF-kB (Zhang et al., 2017). These data are supportive of the hypothesis that excessive ROS is detrimental for chronic low-grade inflammation in vasculature. In Apoe^-/-^ mice, UCP2 also mediates the beneficial effect of AMPK activation against vascular inflammation and atherogenesis (Wang et al., 2011). However, another study showed that hepatic UCP2 may be involved in lipid catabolism in PPARα-treated atherosclerotic Ldlr^-/-^ mice (Srivastava et al., 2006). Since most studies used a global knockout of Ucp2, the role of cell type-specific UCP2 in atherogenesis remains unsolved, and it is unknown whether endothelial UCP2 plays an important role in atherogenesis.

### UCP2 and Other Vascular Diseases

Unlike systemic arterial blood vessels, pulmonary vasculature is very sensitive to metabolic disturbance on glucose oxidation. In fact, Ucp2^-/-^ mice develops spontaneous pulmonary arterial hypertension and vascular remodeling, due to the deficiency of Ucp2 in vascular smooth muscle, which causes mitochondrial membrane hyperpolarization, lowers mitochondrial calcium, and inhibits enzymes such as pyruvate dehydrogenase. This Ucp2^-/-^ phenotype is similar to hypoxic condition that can trigger anti-apoptotic and proliferative signaling pathways leading to arterial remodeling in the lungs (Dromparis et al., 2013; Pak et al., 2013). However, recent experiments in VEcad-Cre (endothelial-specific) Ucp2 deficient mice showed that endothelial Ucp2^-/-^ is accompanied by increased mitophagy and autophagy, decreased mitochondrial content, and apoptotic responses, while these changes exaggerate the increased pulmonary arterial pressure and right ventricle hypertrophy induced by intermittent hypoxia in mice (Haslip et al., 2015). Interestingly, loss-of-function genetic variant of UCP2 in pulmonary arterial hypertensive patients causes resistance to PDK inhibitors, due to the regulation of PDH (the downstream target of PDK) by UCP2-dependent mitochondrial calcium homeostasis (Michelakis et al., 2017). These studies suggest that vascular UCP2 is an important regulator of mitochondrial function in response to hypoxic stress in the development of vascular remodeling.

Mitochondrial function is also critical for cerebrovascular and neuronal function. Acute cerebral ischemia causes mitochondrial dysfunction, oxidative stress, and apoptosis. Indeed, deletion of Ucp2 worsens ischemic damage, suppresses cell-cycle and DNA-repair genes, and increases inflammatory cytokines (Haines et al., 2010), indicating a similar function of Ucp2 in cerebral vasculature as in other organs. Ucp2 overexpression accelerates neurological recovery and reduces apoptosis in response to ischemia (Mattiasson et al., 2003).

The up-to-date information on UCP2 function in vasculature is mainly confined to its protective role against uncontrolled mitochondrial ROS production in endothelial and vascular smooth muscle cells. UCP2 also regulates mitochondrial Ca^2+^ uptake in endothelial cells (Trenker et al., 2007). One study suggested that overexpression of UCP2 might further deteriorates mitochondrial dysfunction due to increased Ca2+ uptake in mouse model of neuro-degenerative disease (Peixoto et al., 2013). Yet whether mitochondrial Ca^2+^ overload induced by pro-inflammatory stimuli when UCP2 expression is up-regulated, whether it is affected by the redox status (mitoROS), whether it contributes to imbalance of ER Ca^2+^ level, which can leads to unfolded protein response, and what is its implication in cardiovascular disease, require more detailed mechanistic study in the future.

## UCP2 and Cardiac Diseases

### Genetic Polymorphism of UCP2 in Coronary Artery Disease

UCP2 expression alters in the settings of coronary artery disease (CAD) and myocardial ischemia. A common polymorphism -866G>A in the promoter region of human *UCP2* has been associated with the risk of CAD. A 10-year prospective cohort study found that the *UCP2*-866A allele homozygosis doubles CAD risk after adjustment for other risk factors (Dhamrait et al., 2004). The increased risk associated with this genotype is linked with the increased oxidative stress and the presence of other risk factors including obesity, hypertension, and diabetes (Dhamrait et al., 2004). The A allele is also associated with increased smoking-induced oxidative stress (Stephens et al., 2008), reduced low density lipoprotein particle size (Hamada et al., 2008), and blunted response to beta-blocker therapy (Beitelshees et al., 2010). Further work demonstrated that diabetic patients in the post-myocardial infarction cohort with *UCP2*-866A allele have poorer survival (Palmer et al., 2009). Moreover, the A allele carriers exhibit decreased UCP2 expression (Beitelshees et al., 2010). These evidences indicate that UCP2 reduces the CAD risk. However, another study showed that the *UCP2*-866A allele is associated with reduced risk of CAD in type 2 diabetic men in a 6-year prospective study (Cheurfa et al., 2008). The reasons for this discrepancy remain unknown, but it may be due to the racial and population differences, as there is also a study failed to establish a linkage between the *UCP2*-866G/A and the risk of CAD in young South African Indian (Phulukdaree et al., 2013). Besides the genotype influencing the expression level, the A55V polymorphism causing the UCP2 dysfunction also increased the risk of cardiovascular events in patients with CAD (Gioli-Pereira et al., 2013). Collectively, *UCP2* polymorphism alters the risk of developing CAD in some populations.

### UCP2 and Myocardial Ischemic Injury

Myocardial ischemic injury is a major clinical problem leading to cardiac dysfunction, in which the oxidative stress and damage play a key role. Accumulating evidences indicate that the expression level of UCP2 is upregulated in the ischemic myocardium, probably in response to the increased oxidative stress during hypoxia either with or without reperfusion (McFalls et al., 2006; Cabrera et al., 2012; Safari et al., 2014a; Colbert et al., 2015). UCP2 protein expression increases in the ischemic left ventricles early after acute myocardial I/R injury, but it is unchanged in the non-ischemic right ventricles of rats. In addition, UCP2 expression increases in swine myocardium during late ischemic preconditioning (Cabrera et al., 2012), and also increases in chronically ischemic swine myocardium (McFalls et al., 2006). Moreover, the expression of UCP2 remains elevated within the myocardium during the early phase after revascularization (Holley et al., 2015). However, treatment with pharmacological agents that target IR injury, including losartan and Ramipril, suppresses UCP2 expression in myocardial tissue (Safari et al., 2014b), possibly due to the inhibition of ROS production. Moreover, a gene knockdown approach demonstrates that UCP2 is required for augmenting the ischemia tolerance of myocardium (McLeod et al., 2005). The cardioprotective effect of SIRT1 is also mediated by UCP2 in IR injury (Deng et al., 2017). UCP2 is often used recently as a protective marker for oxidative stress and mitochondrial injury in animal models of cardiac injury or ischemic insults (Castillo et al., 2018; Quan et al., 2018; Wang et al., 2018) (**Table 2**). Based on these evidences, the upregulation of UCP2 is a response triggered by the change of redox status induced by hypoxia or during early phase after hypoxia a feedback protective mechanism to restore the balance.

In addition to modulating the redox status to reduce tissue damage after ischemia or ischemia/reperfusion, mitochondrial Ca^2+^ homeostasis might also be involved in the function of UCP2 in ischemic myocardium, which is less understood. The protective role of UCP2 can be demonstrated in Ucp2^-/-^ mice, which are particularly susceptible to hypoxic stress (Motloch et al., 2015). Application of a blocker of mitochondrial calcium uniporter (MCU), Ruthenium 360, reduced the rate of cell death by apoptosis (Motloch et al., 2015). Protective roles of UCP3 have also been observed in ischemia and reperfusion of *ex vivo* Ucp3^-/-^ mouse hearts (Ozcan et al., 2013). The isolated heart method has been extensively used to examine whole heart hemodynamics and electrophysiology (Tse et al., 2016a,c). Under I/R conditions, Ucp3^-/-^ hearts showed increased infarct size, poorer recovery of left ventricular systolic function and higher incidence of arrhythmias (Ozcan et al., 2013). This was accompanied by reduced ATP content, higher ROS levels (Ozcan et al., 2013). Moreover, the protective effects of H_2_O_2_ in IR injury were shown to be Ucp3-dependent, where preservation of mitochondrial function was explained by inhibition of mitochondrial permeability transition pore opening via the PI3-kinase/protein kinase B pathway (Chen et al., 2015). However, another study using UCP2 inhibitor genipin and Ucp2^-/-^ mice showed that inhibition of UCP2 attenuates cardiac hypertrophy induced by transverse aortic constriction without affecting blood pressure in mice. Such effect is attributed to enhanced oxygen utilization and ATP synthesis (Ji et al., 2015), perhaps also due to the effect of UCP2 in mediating mitochondrial Ca^2+^ uptake. These discrepancies may be caused by different treatment window, the cause of hypertrophy, and the biological dynamic during the development of hypertrophy, which need to be further examined. Taken together, the compensatory upregulation of UCP2 in ischemic myocardium plays a beneficial effect on I/R injury.

### UCP2 in Cardiomyopathy and Heart Failure

Although UCP2 provides an anti-oxidative effect on myocardial tissue, it also puts the myocardium under a low-energy state through the uncoupling of oxidative phosphorylation. Therefore, UCP2 has potential deleterious effects on failing heart, with a mismatch between energy production and utilization (Neubauer, 2007). Likewise, UCP2 expression was reduced in the doxorubicin- and streptozotocin-induced cardiomyopathy models (Zhang et al., 2012; Hao et al., 2015). However, it was increased in the hearts of animals with β-adrenergic receptor overexpression or following thyroid hormone treatment (Degens et al., 2003; Gaussin et al., 2003). Changes in UCP2 expression could be observed before the overt development of cardiomyopathy, which can be explained by physiological stimulation of UCP2 following adrenergic activation. UCP2 is decreased in both animal models and patients with heart failure (Laskowski and Russell, 2008), allowing a rational approach of overexpressing UCP2 in an attempt to ameliorate heart failure. Indeed, adenovirus-mediated overexpression of UCP2 lowers mitochondrial membrane potential and protects against oxidative stress (Turner et al., 2010). However, caution must be made as increased UCP2 expression also impairs beat-to-beat calcium handling and excitation–contraction coupling without significantly affecting total cellular ATP level (Turner et al., 2010). This is possibly due to the disturbance of UCP2-mediated endoplasmic reticulum Ca^2+^ handling induced by mitochondrial Ca^2+^, meaning that overexpression of UCP2, especially when it is much higher than physiological level, can exacerbate the loss of contractile function of failing heart. This impairment of calcium handling is also potentially arrhythmogenic through the development of cardiac alternans which can increase the susceptibility to reentry (Tse et al., 2016b).

The beneficial effects of UCP2 have also been attributed to metabolic adaptations (Kukat et al., 2014). In a mouse model of premature aging using mtDNA mutations, the mice had reduced food intake and weight loss, which was accompanied by a cardiomyopathic process leading to heart failure. UCP2 was upregulated but this was not associated with either altered proton leak or ROS production. Instead, UCP2 promoted fatty acid metabolism, supporting the metabolic hypothesis and argues against progressive respiratory deficiency.

## Concluding Remarks

The ability of UCP2 to limit mitoROS production has attracted increasing attention and interest among researchers to investigate its functional role in many cardiovascular diseases in which excessive oxidative stress is present. Aside from its uncoupling and its anti-oxidative property, UCP2 also modulates various cellular energetic processes, such as fatty acid oxidation, glycolysis, and mitochondrial calcium uptake, and possibly thermoregulation, but these are relatively less well characterized when increasing attention has been given on the intrinsic cellular metabolism of vascular cells and the implication of its dysregulation in the context of cardiovascular disease. Whether these mechanisms reflect disease phenotype when cell is exposed to different stressors and damaging factors remain incompletely understood. A recent study showed that UCP2 is responsible for DRP1-dependent mitochondrial fission upon glucose load in SF1 neurons of ventromedial nucleus, and this process is important for regulating peripheral glucose homeostasis (Toda et al., 2016). The regulation of mitochondrial fission/fusion by UCP2 may also be important in other tissues that can be further explored. In addition, studies of UCP2 function in different organs have demonstrated conflicting results. However, one needs to be aware that the approach of using artificial overexpression or transgene has its limitation in implicating the homeostasis and dysregulation of cell or tissue. Therefore, cell type-specific modification of UCP2 expression/function may represent a better approach to elucidate the mechanisms by which UCP2 governs mitochondrial function and ROS production, and how the organism as a whole responds to the dynamics, especially in certain types of disease where multiple cell types/tissues are involved.

## Author Contributions

XT and SM conceived and drafted the work. GT and WW revised the work and provided critical comments. YH conceived the work and revised critically.

## Conflict of Interest Statement

The authors declare that the research was conducted in the absence of any commercial or financial relationships that could be construed as a potential conflict of interest.
